# Oridonin Enhances Radiation-Induced Cell Death by Promoting DNA Damage in Non-Small Cell Lung Cancer Cells

**DOI:** 10.3390/ijms19082378

**Published:** 2018-08-13

**Authors:** Hyejin Park, Ye Ji Jeong, Na-Kyung Han, Joong Sun Kim, Hae-June Lee

**Affiliations:** 1 Division of Radiation Biomedical Research, Korea Institute of Radiological and Medical Sciences, Seoul 01812, Korea; jhp13@hanmail.net (H.P.); brightwisdm0914@gmail.com (Y.J.J.); gmxvz@hanmail.net (N.-K.H.); 2 K-Herb Research Center, Korea Institute of Oriental Medicine, Daejeon 34054, Korea; centraline@kiom.re.kr

**Keywords:** oridonin, radiation, sensitization, ROS, apoptosis

## Abstract

Although many attempts have been made to improve the efficacy of radiotherapy to treat cancer, radiation resistance is still an obstacle in lung cancer treatment. Oridonin is a natural compound with promising antitumor efficacy that can trigger cancer cell death; however, its direct cellular targets, efficacy as a radiosensitizer, and underlying mechanisms of activity remain unclear. Herein, we report that oridonin exhibits additive cytotoxic and antitumor activity with radiation using the H460 non-small cell lung cancer cell lines. We assessed the effect of oridonin by proliferation, clonogenic, reactive oxygen species (ROS) production, DNA damage, and apoptosis assays. In vitro, oridonin enhanced the radiation-induced inhibition of cell growth and clonogenic survival. Oridonin also facilitated radiation-induced ROS production and DNA damage and enhanced apoptotic cell death. In vivo, the combination of oridonin and radiation effectively inhibited H460 xenograft tumor growth, with higher caspase-3 activation and H2A histone family member X (H2AX) phosphorylation compared with that of radiation alone. Our findings suggest that oridonin possesses a novel mechanism to enhance radiation therapeutic responses by increasing DNA damage and apoptosis. In conclusion, oridonin may be a novel small molecule to improve radiotherapy in non-small cell lung cancer.

## 1. Introduction

Lung cancer is a leading cause of cancer-related deaths worldwide. Radiotherapy is an important treatment for unresectable advanced human lung cancers, as well as an adjuvant therapy after surgery and in palliative treatment. It is used at every stage of clinical progression, with both non-small cell lung cancer (NSCLC) and small cell lung cancer (SCLC) forms of the disease [1]. According to epidemiological studies, over 60% of patients with NSCLC have been treated with radiotherapy [2,3]. However, lung cancer radiotherapy is far from ideal due to problems associated with radiation resistance of the cancerous cells and severe cytotoxicity against noncancerous cells [4]. Despite recent advances in the delivery of lung cancer radiotherapy, most patients relapse and show poor survival [5,6]. Therefore, it is necessary to develop new strategies to improve the efficacy of this treatment procedure.

Radiation resistance is a major impediment to the success of cancer therapy. There have been many attempts to reduce radioresistance to lung cancer treatment with the use of radiation sensitizers, which have the potential to overcome resistance and improve treatment outcomes. There have been many clinical trials examining the efficacy of enhancing radiotherapy; many of these trials tested agents that were cytotoxic chemotherapies such as paclitaxel and cisplatin [7,8]. In addition, protein Kinase B (PKB, otherwise known as AKT), mammalian target of rapamycin (mTOR), and checkpoint kinase 1 (Chk1) have been extensively studied as potent radiosensitizers, but their benefits are limited due to their broad biological activities and potential side effects [9,10,11]. Due to a critical need for the discovery and development of new radiation enhancers, many investigations are ongoing on novel classes of small molecule radiation sensitizers that have high efficacy and low toxicity, including those targeting survivin [12,13].

Oridonin is a natural diterpenoid compound that can be isolated from *Rabdosia rubescens* (*Isodon rubescens*) and other plants in the genus *Isodon*; these plants are used in Chinese and Japanese traditional medicines for the treatment of various human diseases [14,15]. Oridonin has been reported to exhibit potent anticancer activity both in vitro and in vivo against various cancer cells, including human gastric cancer [16,17], colorectal cancer [18], breast cancer [19], and leukemia [20] cells. Although many proteins, including extracellular signal-regulated kinase (ERK) [21], BCL2-Associated X Protein (Bax)/B-cell lymphoma-extra large (Bcl-xL) [22], and nuclear factor (NF)-κB [23,24] have been found to be involved in the anticancer activity of oridonin, its therapeutic effects remain largely unknown. Specifically, a radiation sensitizing effect of oridonin has not been established for cancer treatment.

The aim of this study was to investigate oridonin as an effective adjuvant for radiotherapy and explore its mechanism of action on human lung cancer cells. We found that oridonin could sensitize H460 NSCLC cells (both cultured and as xenograft tumors in mice) to radiation-induced cell death, most likely by increasing production of reactive oxygen species (ROS), DNA damage, and apoptosis.

## 2. Results

### 2.1. Oridonin Inhibits H460 Lung Cancer Cell Growth

**Figure 1:**
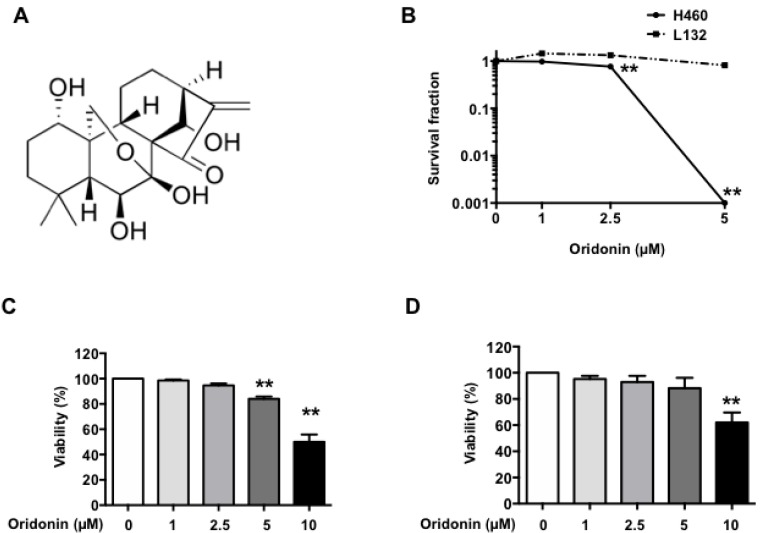
The cytotoxic effect of oridonin on human lung cancer cells and non-cancer cells. (**A**) Molecular structure of oridonin; (**B**) survival fractions obtained by colony forming assay. H460 cells and L132 cells were incubated for 7 days in the presence of 0 µM to 5 µM oridonin. MTT (3-(4,5-dimethylthiazol-2-yl)-2,5-diphenyltetrazolium bromide) assay in H460 cells (**C**) and L132 cells (**D**). Cells (5 × 10^3^) were seeded into 24-well plates and incubated for 48 h in the presence of 0 µM to 10 µM oridonin. All experiments were independently performed three times. The data are shown as means ± SEM; ** *p* < 0.01 versus 0 µM oridonin.

Oridonin (Figure 1A) is a multifunctional drug that demonstrates powerful anticancer effects. Accordingly, we determined the effects of oridonin alone on H460 human lung cancer cells and L132 human lung epithelial cells using clonogenic survival assays (Figure 1B). Five µM of oridonin showed no colony formation in H460 cells while L132 cells were not affected by oridonin alone up to 5 µM. Next, we examined the growth-inhibition effect of oridonin using an MTT (3-(4,5-dimethylthiazol-2-yl)-2,5-diphenyltetrazolium bromide) assay. The cells were treated with different concentrations of oridonin (1, 2.5, 5, and 10 µM) for 48 h, and the cell viability was measured. Oridonin inhibited H460 cell proliferation at 5 µM to 10 µM (Figure 1C). However, oridonin also showed cytotoxic effects on L132 human lung epithelial cells at 10 µM (Figure 1D). To reduce potential cytotoxicity toward normal cells, we restricted the oridonin treatment of the H460 cells to 5 µM for subsequent studies.

### 2.2. Oridonin Enhances Radiation Effects on Lung Cancer Cells

**Figure 2:**
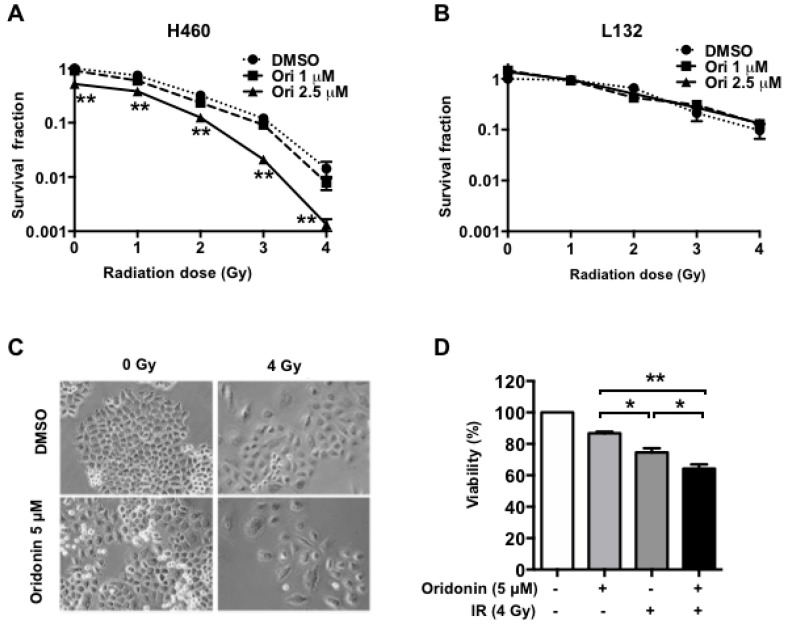
Effect of oridonin on sensitivity of H460 cells to radiation. (**A**) Survival fractions obtained by colony forming assay in H460 cells (**A**) and L132 cells (**B**). ** *p* < 0.01; *p*-values represent two-way ANOVA results. (**C**) Representative images of cultured H460 cells in the presence of oridonin (5 µM) and/or radiation (IR, 4 Gy). (**D**) H460 cell viability was measured using an MTT assay. The data are presented as means ± SEM (*n* = 3); * *p* < 0.05 and ** *p* < 0.01. Three independent experiments were carried out. DMSO, dimethyl sulfoxide vehicle.

Next, we conducted clonogenic survival assays to determine the effect of oridonin on H460 cells’ response to radiation. The H460 cells were treated with 1 µM or 2.5 µM of oridonin for 1 h prior to radiation and then were exposed to different doses of gamma rays. The assessment of colony formation showed that the surviving cell fraction with combined oridonin and radiation treatment was significantly decreased compared to that with radiation alone (two-way ANOVA test with Tukey’s multiple comparison, *p* < 0.01, Figure 2A). However, oridonin did not affect radiation response of L132 cells (Figure 2B). To further investigate the combination effect of oridonin and radiation, cell viability was examined with oridonin and/or radiation using an MTT assay. Treatment of the cells with 5 µM oridonin or 4 Gy of gamma irradiation inhibited H460 cell growth by 12% and 23%, respectively; combining the two treatments inhibited their growth by 33% (Figure 2C,D). These data indicate that oridonin enhances the cytotoxic effect of radiation in H460 lung cancer cells.

### 2.3. Oridonin Enhances Radiation-Induced ROS Production

**Figure 3:**
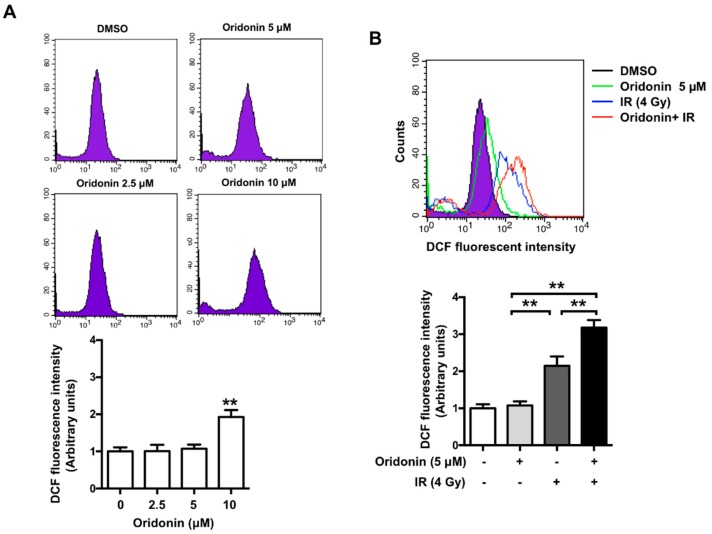
The effect of oridonin on radiation-induced ROS accumulation. (**A**) Cellular reactive oxygen species (ROS) levels were determined by DCFH-DA staining 48 h after treatment with oridonin (0 μM to 10 µM). DCFH-DA fluorescence was measured by flow cytometry. (**B**) Univariate histogram plots represent DCF mean fluorescence of control (vehicle, 0.05% DMSO), oridonin (5 µM), IR (4 Gy), and a combination of oridonin and IR (**upper panel**). DCF fluorescence was quantified and values were normalized to the mean fluorescence of the control (**lower panel**). Values are the average of three independent experiments (means ± SEM; ** *p* < 0.01). DCFH-DA, 2′,7′-dichlorodihydrofluorescein diacetate; DMSO, dimethyl sulfoxide.

Oridonin has been reported to exhibit antitumor effects via ROS production [25]. Therefore, to explore further the mechanisms by which oridonin increases the radiation sensitivity of lung cancer cells, we investigated whether oridonin affects ROS production. We measured ROS production in H460 cells with increasing concentrations of oridonin from 2.5 μM to 10 µM. There was a significant increase in ROS production by ~2-fold at 10 µM oridonin, but concentrations ≤5 µM had no effect on ROS production (Figure 3A). Compared with that of irradiation alone, co-treatment with 5 µM oridonin significantly enhanced radiation-induced ROS production (*p* < 0.01, Figure 3B).

### 2.4. Oridonin Accelerates Radiation-Induced DNA Damage

**Figure 4:**
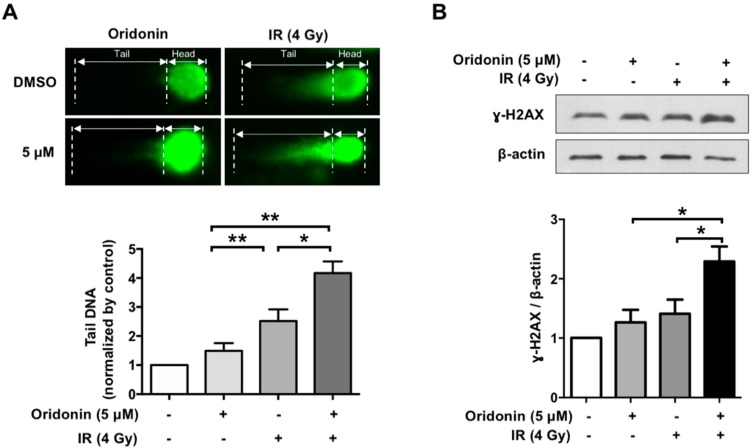
Effect of oridonin on radiation-induced DNA damage. (**A**) Representative images and quantification of DNA comet assays. Comet tail sizes were measured by OpenComet, an ImageJ program, and normalized to the tail intensity of the DMSO control; (**B**) phospho-histone H2AX (γ-H2AX) protein levels were analyzed by Western blot. Ten µg of protein was loaded per lane (**upper panel**). Band intensity was quantified by ImageJ and normalized to control levels (white bar, **bottom panel**). Data are representative of more than three independent experiments and values are expressed as means ± SEM; * *p* < 0.05 and ** *p* < 0.01. DMSO, dimethyl sulfoxide.

As it is known that an increase in ROS levels can result in DNA damage [26,27], we determined the effect of oridonin on radiation-induced DNA damage using a comet assay. This DNA damage was determined by measurement of the DNA comet tail, photographed under a microscope, using OpenComet (the open-source software in ImageJ) [28]. Oridonin treatment alone induced mild DNA damage at 5 µM (1.5-fold, *p* = 0.69), while 10 μM oridonin significantly increased DNA damage (2.14-fold, *p* = 0.02) compared to that of the 0.05% DMSO control. Compared with that of both oridonin and IR individual treatments, the combination of 5 µM oridonin and irradiation showed a significant increase in the size of the DNA comet tail in the treated H460 cells (Figure 4A). Additionally, we evaluated the effect of oridonin on phospho-histone H2AX (γ-H2AX), a common marker of DNA damage. Five µM of oridonin alone did not alter γ-H2AX levels, but oridonin in combination with IR significantly increased γ-H2AX content (*p* < 0.05, Figure 4B).

### 2.5. Oridonin Increases Radiation-Induced Apoptotic Cell Death

**Figure 5:**
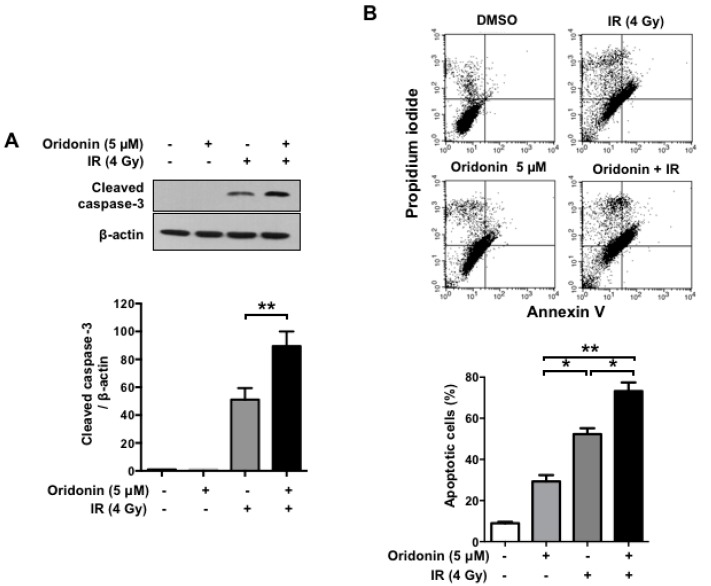
Effect of oridonin on radiation-induced cleavage of caspase-3 and cell death. Cells were treated with oridonin (5 µM) and/or IR for 72 h. (**A**) Representative Western blots (**upper panel**) and plotted, quantified data (**lower panel**) for cleaved caspase-3; (**B**) cells were stained with Annexin V-FITC/PI prior to flow cytometry analysis. Apoptotic cells were estimated by the sum of the **upper left**, **upper right**, and **lower right** quadrants in each dot plot. Plotted values represent the percentages of apoptotic cells. All plotted data represent the means ± SEM of three independent experiments * *p* < 0.05 and ** *p* < 0.01. FITC, fluorescein isothiocyanate; PI, propidium iodide.

To explore the mechanisms by which oridonin enhances the radiation sensitivity of lung cancer cells, we investigated whether it increased IR-induced cleavage of caspase-3. Western blot analysis showed that the combination of oridonin and irradiation profoundly increased the cleavage of caspase-3 compared to that of irradiation alone (*p* < 0.01, Figure 5A). According to the caspase activation time, we also conducted flow cytometry analysis to evaluate apoptotic cell death induced by oridonin and/or IR treatment in H460 cells using an Annexin V-fluorescein isothiocyanate (FITC) apoptosis detection kit (Figure 5B). Flow cytometry revealed that oridonin and IR alone induced apoptotic cell death in 29% and 52% of the cells, respectively; however, their combination significantly increased the proportion of apoptotic cells to 73% (*p* < 0.05). These findings suggest that co-treatment with oridonin enhances radiation-induced apoptotic cell death.

### 2.6. Oridonin Enhances Radiation-Induced Tumor Growth Inhibition

**Figure 6:**
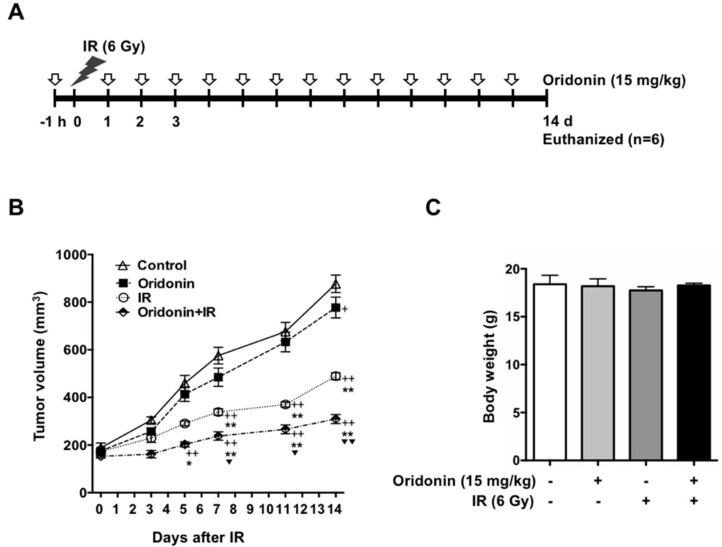
Effect of oridonin on radiotherapy. (**A**) Schema of the timeline for oridonin and irradiation treatment. Daily oridonin (15 mg/kg, intraperitoneal injection) was administered up to the indicated time points pre- and post-irradiation (6 Gy); (**B**) growth curves of H460 tumors following oridonin and/or IR treatment. (**C**) Mean body weights of experimental groups at study termination. The data represent the means ± SEM of six mice per group. ^+^ *p* < 0.05 and ^++^ *p* < 0.01 compared to control; * *p* < 0.05 and ** *p* < 0.01 compared to oridonin; **^▼^** *p* < 0.05 and **^▼▼^** *p* < 0.01 compared to IR.

**Figure 7:**
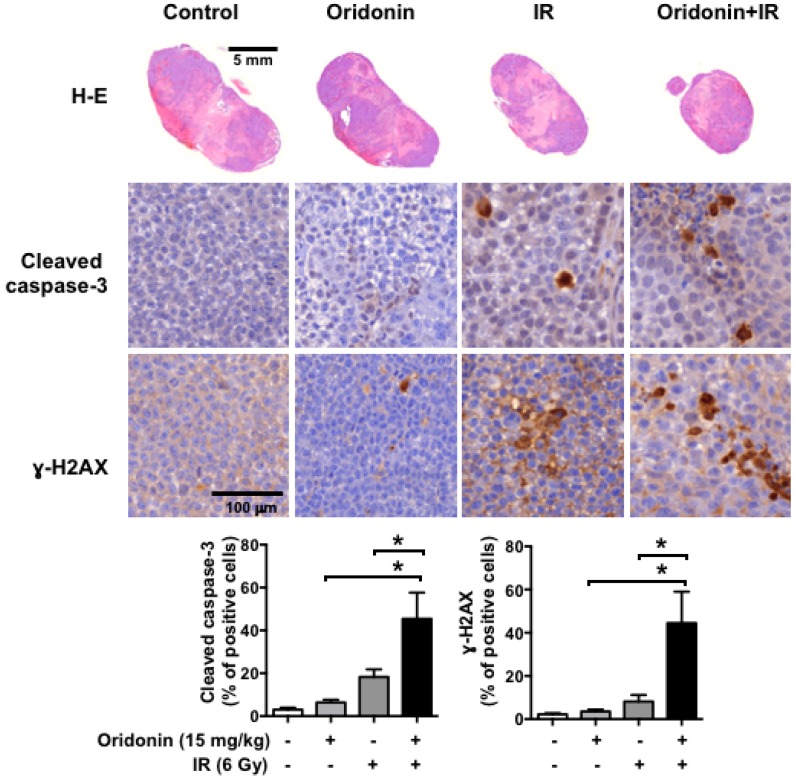
Histopathological analysis of H460 tumors following combination treatment with oridonin and radiation. Hematoxylin and eosin (H-E) staining and immunohistochemistry for cleaved caspase-3 and γ-H2AX were performed on tumors harvested at 14 days after IR. Representative images of H-E-stained tumors (**upper images**) and cleaved caspase-3- and γ-H2AX-positive cells (**middle images**, brown staining) and quantification of cleaved caspase-3 and γ-H2AX-positive staining with six mice in each group (**lower plots**, means ± SEM) are shown; * *p* < 0.05.

To investigate the additive effect of oridonin in vivo, we established an H460-bearing tumor model. Once the tumors reached 150 mm^3^, the mice were treated with radiation (6 Gy) and/or oridonin (15 mg/kg) as shown in Figure 6A. We observed significant inhibition of tumor growth by co-treatment with oridonin and radiation after 7 days compared to that with radiation and oridonin alone. At 14 days, when the animal experiment was terminated, we determined the inhibitory effect of each treatment by analyzing tumor volumes and comparing them to the control tumor volumes. Oridonin alone (15 mg/kg) showed a weak inhibitory effect on tumor growth (up to 11%, *p* < 0.05). IR alone (6 Gy) led to 44% growth inhibition when compared to that of control (*p* < 0.05), while the combination of oridonin and IR decreased tumor volume by 65% (*p* < 0.01 versus control). Combination treatment of oridonin and IR significantly reduced tumor volume compared to oridonin alone (*p* < 0.01) and IR alone (*p* < 0.01) (Figure 6B). IR treatment alone led to a slight decrease in body weight; however, there was no significant difference in body weights among the groups at the end of treatment (Figure 6C). Immunohistochemical analysis showed that co-treatment with oridonin increased cleaved caspase-3 and γ-H2AX levels up to 2.2-fold (*p* < 0.05) and 2.6-fold (*p* < 0.05), respectively, compared to the values with IR alone (Figure 7). These findings suggest that the mechanism of oridonin-mediated enhancement of radiation’s antitumor effect involves increased DNA damage and apoptotic cell death.

## 3. Discussion

Radiotherapy is an important modality in lung cancer patients during the course of cancer treatment both as a curative modality and for palliation. However, tumor radioresistance and toxic side effects toward normal tissue, which impede dose escalation, are major obstacles to the success of radiation therapy [29,30]. Thus, strategies for optimizing the response of cancer to increase the therapeutic efficiency of radiotherapy are needed. There is an increasing interest in combining radiation and natural compounds to enhance the efficacy of radiotherapy. The use of natural products as antitumor agents or radiosensitizers for the management of human cancers is an attractive idea because they are readily available and exhibit little or no toxicity [31,32,33]. Oridonin, a natural tetracyclic diterpenoid compound, has well-known potent anticancer activity against a wide range of cancer cell types, including prostate [34] and breast cancers [19] and acute leukemia [20]. It has been reported that oridonin elicits an antiproliferative effect on lung cancer cell lines in vitro and in vivo [35,36]. However, the effects of oridonin as an adjuvant of radiation on lung cancer cells remain poorly understood. In this study, we found a novel potential of oridonin to sensitize the anticancer effect of radiation via increases in ROS accumulation, DNA damage, and apoptosis in H460 cells. To the best of our knowledge, this is the first report to support the potential beneficial effect of oridonin to improve responses to radiotherapy in NSCLC patients.

Many recent studies have focused on the antitumor effects of oridonin, and its derivatives have been developed to be used in combination with other anticancer drugs. Guo et al. reported that oridonin synergizes the anti-leukemia effect of imatinib via the LYN Proto-Oncogene (LYN)/mTOR pathway [37]. Zhang et al. also reported that oridonin treatment could overcome cisplatin-resistance in human acute leukemia cells [38]. Wu et al. reported that an analog of oridonin, named CYD-6-28, effectively suppressed triple-negative breast cancer cell growth via induction of death receptor 5 [39]. Therefore, our findings and those from other investigators strongly support the potential of oridonin as an anticancer agent.

An ideal radiosensitizer enhances the ability of radiation to kill tumor cells while not altering the radioresponse of normal tissues [40]. We investigated the ability of oridonin, a natural small molecule, to act as an effective adjuvant in human non-cancer cells. As previously reported, we found that oridonin has a potent anticancer effect that inhibits the proliferation and clonogenic ability of cancer cells. However, we also identified cytotoxicity against non-cancerous cells that was not considered in previous studies. Specifically, oridonin demonstrated cytotoxicity against both normal lung epithelial cells and lung cancer cells. To diminish the toxic side effects of oridonin, we used a lower concentration of oridonin (5 µM), which showed a weaker anticancer effect compared to that in previous studies, to observe its beneficial effects on radiotherapy. Five µM of oridonin alone did not alter ROS production and DNA damage in the comet assay, but still showed mild effects on growth inhibition and cell death of the H460 cells. However, notably, the combination of 5 µM oridonin and radiation greatly enhanced ROS production, DNA damage, and apoptotic cell death of H460 cells. In addition, H460 tumors were sensitized to the effect of radiation by oridonin treatment, which was assessed by the inhibition of tumor growth (i.e., tumor volume). Although the dose of oridonin used in this study (15 mg/kg) is a low to moderate dose compared to that used in previous studies (7.5 mg/kg to 40 mg/kg) [41,42,43], the combination of oridonin and radiotherapy significantly inhibited tumor growth in the experimental time period. Our results suggest carefully considering dose reduction to minimize side effects while promoting therapeutic efficacy. Therefore, further studies will be needed to optimize the dosage and assess the toxicity of oridonin in various types of cancer.

In the present study, we demonstrated that oridonin enhanced the therapeutic effect of radiation by enhancing cancer cell death via acceleration of DNA damage and ROS production. Our results suggest the potential of oridonin as a novel enhancer of radiotherapy.

## 4. Materials and Methods

### 4.1. Cells and Treatments

The NCI-H460 NSCLC cell line was cultured in RPMI medium (WELGENE, INC., Gyeongsan-si, Gyeongsangbuk-do, Korea) with 10% fetal bovine serum (FBS, 35-015-CV, CORNING, Manassas, VA, USA) and 1% Gibco^®^ antibiotic-antimycotic (15240-062, Thermo Fisher Scientific, Waltham, MA, USA) at 37 °C in a 5% CO_2_ humidified incubator. The cells were seeded at the indicated numbers and incubated overnight before treatment with various concentrations of oridonin and doses of IR. Oridonin was purchased from Selleck Chemicals (S2335, Houston, TX, USA).

### 4.2. MTT Assay

H460 cells and L132 cells (5 × 10^3^) were seeded into 24-well plates and incubated overnight. Cells were incubated in medium with the indicated concentration of oridonin for 1 h prior to radiation and then the culture medium was not replaced for 48 h. After incubation, MTT stock solution (5 mg/mL) was added at 10% of the culture medium volume, and the cells were incubated for 2 h at 37 °C. Then, the medium was discarded and dimethyl sulfoxide (DMSO) (200 µL) was added to each well and incubated for 10 min to dissolve the insoluble formazan precipitate. The solution was then transferred to a 96-well plate and its absorbance read at 560 nm.

### 4.3. Clonogenic Aassay

The cells were seeded in 60-mm dishes according to radiation dose (0 Gy 100 cells, 1 Gy 200 cells, 2 Gy 500 cells, and 4 Gy 2000 cells) and incubated overnight. Then, cells were incubated in medium with the indicated concentration of oridonin for 1 h prior to radiation. The cells were then exposed to radiation using ^137^Cs as a radiation source (BIOBEAM GM 8000, Gamma Service Medical GmbH, Leipzig, Germany). Culture medium was not replaced after radiation, but fresh medium containing oridonin was added every 2 or 3 days during the colony-forming unit (CFU) assay. At seven days after radiation, the dishes were washed with phosphate-buffered saline (PBS) and incubated with 0.5% crystal violet in 5% neutral-buffered formalin. Then, the dishes were washed and the colonies were counted.

### 4.4. Determination of ROS Production

Cellular ROS levels were estimated using 2,7-dichlorodihydrofluorescein diacetate (DCFH-DA) purchased from Cayman Chemical (85155, Ann Arbor, MI, USA). The cells were trypsinized and mixed with 1% FBS in PBS containing 10 µM DCFH-DA for 15 min, while protecting it from light. The fluorescent cells were analyzed on a FACSCalibur^TM^ flow cytometer (Becton-Dickinson, San Diego, CA, USA).

### 4.5. Comet Assay

The CometAssay^TM^ (4250-050-K, TREVIZEN, Gaithersburg, MD, USA) was used for DNA damage assessment. Cells (1 × 10^5^) were collected in 50 µL ice-cold PBS. Ten volumes of completely melted low-melting agarose (LMA) was mixed with 10 volumes of suspended cells; 75 µL cell/agarose mixture was spread on the comet slide and incubated at 4 °C for 30 min to solidify the agarose. After congelation, the slides were incubated in lysis solution (4250-050-01, TREVIZEN) at 4 °C for 60 min. Then, the slides were moved carefully into alkaline solution (pH > 13) and incubated at room temperature for 30 min. For neutralization, the slides were washed once in 1 × Tris-borate-EDTA (TBE) buffer and electrophoresis was performed at 31 V for 40 min. The slides were washed with 70% ethanol and air dried after removing excess liquid. Fifty microliters of SYBR^®^ Green I were placed on each sample, and the slides were analyzed by fluorescence microscopy. The signals of the tail DNA of 50 cells to 80 cells from five high magnification fields (×100) were analyzed to estimate DNA damage in each group. The values were measured using OpenComet, the open-source software tool, in ImageJ software (version 1.52, https://imagej.nih.gov/ij, National Institutes of Health, Bethesda, MD, USA).

### 4.6. Western Blots

The cells (2 × 10^4^) were seeded in 60-mm dishes and incubated for 72 h in RPMI. The cells were trypsinized and lysed in 100 µL of ice-cold radioimmunoprecipitation assay (RIPA) lysis buffer (25 mM Tris–HCl, pH 7.4, 150 mM NaCl, 1% NP-40, 0.5% sodium deoxycholate, 0.5% SDS, 1 mM Na_3_VO_4_, 5 mM NaF, and 1 mM phenylmethylsulfonyl fluoride). The cell suspension was sonicated and incubated on ice for 30 min. After centrifugation at 13,000 rpm for 10 min, the supernatants were collected and protein concentrations were quantified using the bicinchoninic acid (BCA) method (23227, Pierce Biotechnology, Inc., Waltham, MA, USA). For Western blot analysis, proteins (60 µg) were electrophoresed on SDS-polyacrylamide gels and transferred to nitrocellulose membranes. The specific antibodies were cleaved caspase-3 (1:1000, #9661, Cell Signaling Technology, Inc., Danvers, MA, USA), γ-H2AX (1:3000, 05-636, Merck KGaA, Darmstadt, Germany), and β-actin (1:5000, sc-47778, Santa Cruz Biotechnology, Inc., Dallas, TX, USA).

### 4.7. Apoptotic Cell Death by Flow Cytometry

The Annexin V-FITC Apoptosis Detection Kit (K101-25, BioVision Incorporated, Milpitas, CA, USA) was used for detecting apoptotic cell death. The cells were seeded (1 × 10^4^ per well) into 6-well plates and incubated with various concentrations of oridonin before irradiation (IR). The cells were exposed to IR (4 Gy) and incubated for 72 h. The cells were then washed with cold PBS and resuspended in 500 µL of 1× binding buffer containing 5 µL of annexin-FITC and 5 µL of propidium iodide. After mixing, the apoptotic cells were analyzed immediately using the FACSCalibur^TM^ flow cytometer (Becton-Dickinson).

### 4.8. Animal Experiments

All protocols in this study were approved by the Institutional Animal Care and Use Committee of the Korean Institute of Radiological and Medical Sciences (IACUC permit number: KIRAMS2017-0007, 15 February 2017). Six-week-old female BALB/c nude mice were purchased from Orient Bio Inc. (Seongnam-si, Gyeonggi-do, Korea). The animals were maintained at 23 ± 2 °C with humidity of 50 ± 5%, lighting cycle of 08:00 to 20:00, and 13 to 18 air changes per hour. The H460 cells were cultured in RPMI containing 10% FBS and 1% antibiotic/antimycotic at 37 °C in a 5% CO_2_ humidified incubator. A xenograft tumor was created by subcutaneous injection of 1 × 10^6^ cells to the right hind leg. When the tumor volume reached 100 mm^2^ to 120 mm^2^, oridonin treatment (15 mg/kg) was initiated by intraperitoneal injection one hour before IR treatment. Each mouse was anesthetized with tiletamine/zolazepam (Zoletil 50^®^, Virbac Korea, Seoul, Korea) and exposed to 6 Gy of IR using an X-RAD 320 system (Precision X-ray, Inc., North Branford, CT, USA) at 250 kV and 10 mA with 420 mm of aluminum shielding, resulting in a dose rate of 2 Gy/min. The mice were irradiated only in the hind leg region, using a 2 cm × 2 cm field size that included the tumor. Afterward, oridonin was injected daily until 14 days, when the mean tumor volume reached approximately 1000 mm^2^ in the control group. Tumor tissue was then harvested from each mouse for histological analysis.

### 4.9. Immunohistochemistry and Quantification

The tumor tissue was fixed in 10% formaldehyde and embedded in paraffin wax. For histological experiments, the embedded tissues were sectioned at 5 µM, mounted on slides, deparaffinized, and rehydrated by graded ethanol washes. For antigen retrieval, the sections were boiled in citrate buffer (pH 6.0). Immunohistochemistry was performed using a Vectastain^®^ Elite ABC Kit (Vector Laboratories Inc., Burlingame, CA, USA) following the manufacturer’s protocol. The sections were incubated with anti-cleaved caspase-3 (1:100, #9661, Cell Signaling Technology, Inc.), anti-γ-H2AX (1:100, 05-636, Merck KGaA), or anti-Ki-67 (1:200, DRM004, Acris Antibodies, Herford, Germany) antibodies at 4 °C overnight and then washed with PBS containing 0.05% Triton X-100. The sections were incubated with horseradish peroxidase-conjugated secondary antibody for 30 min and counterstained with hematoxylin. The percentage of cleaved caspase-3 and γ-H2AX positive cells were quantified at five randomly selected fields at 400× magnification.

### 4.10. Statistical Analysis

All experimental data are shown as the means ± standard errors of the mean (SEM). The data were analyzed with one-way analysis of variance (ANOVA) followed by Tukey’s post hoc tests or two-way ANOVA using GraphPad Prism version 7.0 software (GraphPad Software, Inc., La Jolla, CA, USA). *p*-values less than 0.05 indicated statistical significance.
